# Expanding Workforce Capacity to Care for Older Veterans: The VA GRECC Interprofessional Training Experience

**DOI:** 10.1093/geroni/igaa057.010

**Published:** 2020-12-16

**Authors:** Kathryn Nearing, Sumathi Misra, Katharina Echt

**Affiliations:** 1 University of Colorado Anschutz Medical Campus, Aurora, Colorado, United States; 2 Veterans Administration, Nashville, Nashville, Tennessee, United States; 3 Department of Veterans Affairs, Atlanta, Georgia, United States

## Abstract

The Veterans Health Administration (VHA) contributes more to training healthcare professionals in geriatrics/gerontology than any other entity nationally, with the Geriatric Research Education and Clinical Center (GRECC) network serving as a leader in geriatric-/gerontology-specific interprofessional education. The Associated Health Training (AHT) Program is supported by all GRECCs, training ~427 trainees annually (FY17). Each AHT program brings together a diverse array of trainees – the specific constellation of disciplines unique to each GRECC based on local capacity/expertise. Common to all programs is intentional interprofessional training, aligned with Interprofessional Education Collaborative (IPEC) competencies. In 2016, the VA Office of Academic Affiliations (OAA) administered a 22-item survey to characterize the depth and breadth of geriatric-/gerontologic-specific interprofessional education across GRECCs. Questions explored how AHT programs addressed each of the four IPEC competency domains. Responses were de-identified; at least 2 coders independently applied directed coding to responses. Across GRECCs (n=18), 323 interprofessional training activities were coded, of which 9% were didactic; 27%, clinical; and, 63%, combination. Interprofessional education activities were integrated with profession-specific curricula (65.3%) or featured as part of GRECC-specific core curricula (5.4%). GRECC AHT interprofessional programs involved an average of 11 disciplines. GRECC AHT programs provide a vital infrastructure for building workforce capacity through robust, interprofessional training that engages diverse disciplines across a variety of care settings representing the continuum of care for older Veterans. GRECC and OAA efforts are critical to enhancing the quality, and expanding the capacity, of this workforce to meet increasing needs for patient-directed, team-based care for older adults.

